# Advancements in Nutritional Strategies for Gestational Diabetes Management: A Systematic Review of Recent Evidence

**DOI:** 10.3390/jcm13010037

**Published:** 2023-12-20

**Authors:** Juan Carlos Sánchez-García, Ines Saraceno López-Palop, Beatriz Piqueras-Sola, Jonathan Cortés-Martín, Elena Mellado-García, Inmaculada Muñóz Sánchez, Raquel Rodríguez-Blanque

**Affiliations:** 1 Research Group CTS-1068, Andalusia Research Plan, Junta de Andalucía, 18014 Granada, Spain; jsangar@ugr.es (J.C.S.-G.); bpiquerassola@gmail.com (B.P.-S.); e.elenamellado@go.ugr.es (E.M.-G.); rarobladoc@ugr.es (R.R.-B.); 2 Department of Nursing, Faculty of Health Sciences, University of Granada, 18071 Granada, Spain; inesaraceno99@correo.ugr.es; 3 Virgen de las Nieves University Hospital, 18014 Granada, Spain; 4 Costa del Sol Health District, 29640 Fuengirola, Spain; 5 “La Chana” Health Center, Granada Health District, 18013 Granada, Spain; inmams1@correo.ugr.es; 6 San Cecilio University Hospital, 18071 Granada, Spain

**Keywords:** gestational diabetes, pregnant, nutrition, diet, eating, food

## Abstract

Gestational diabetes mellitus (GDM) is defined as hyperglycaemia first detected at any time during pregnancy with values lower than those determined by the WHO for diabetes diagnosis in adults. This pathology, with a worldwide prevalence of 13.4%, causes significant maternal and foetal risks. The first line of treatment consists of maintaining normo-glycaemia through an adequate diet and lifestyle changes. The aim is to synthesize the scientific evidence updating the nutritional recommendations for the effective management of GDM. A systematic review of the scientific literature was conducted following the PRISMA guidelines. Randomized clinical trials published within the last five years and providing information on nutritional recommendations to achieve an effective management of gestational diabetes were selected. The databases searched were PubMed, the WOS Core Collection, SCOPUS, and CINAHL, using the MeSH terms: “Diabetes, Gestational”; “Nutrition Assessment (nutrition*)”; “Diet”; “Eating”; and “Food”; with the Boolean operators “AND” and “OR”. The PEDro scale (Physiotherapy Evidence Database) was used to assess the scientific quality of the studies, with a mean score of 8.9, indicating an average good scientific quality. Results: A total of 809 papers were collected, of which, after applying the inclusion and exclusion criteria, 14 randomized clinical trials were selected. Probiotic supplementation and co-supplementation with vitamin D have been found to be the most beneficial options for both mothers with GDM and neonates, but the most effective regimens are not known. Diets enriched with extra virgin olive oil (EVOO) and oat bran, as well as some recommendations focused on carbohydrates also seem effective, as well as diets designed for this group of women with GDM such as “CHOICE”. Conclusions: Although there are numerous proposals that have been published in recent years focused on the diet of women with GDM in order to improve their results and those of their children, it is the supplementation with probiotics and the co-supplementation with vitamin D that is most agreed upon as beneficial; however, more research is needed into which protocols are most effective. Other proposals that could also be beneficial should be further studied.

## 1. Introduction

According to information presented in the document “Classification and Diagnosis of Diabetes: Standards of Care in Diabetes—2023”, the most accurate term to describe hyperglycaemia occurring during pregnancy and diagnosed for the first time in the second or third trimester is “gestational diabetes mellitus” (GDM) [1]. It is crucial to note that gestational hyperglycaemia may also result from pregestational diabetes or diabetes in pregnancy (DIP) [2]. Pregestational diabetes refers to diabetes, either type I or II, diagnosed prior to pregnancy [3]. DIP is typically identified in the first trimester, with pregnant women meeting the diagnostic criteria for non-pregnant adult-onset diabetes according to the World Health Organization (WHO) [4]. Consequently, these individuals should be classified as pre-diabetic pregnant women and treated accordingly [1]. Gestational diabetes, on the other hand, is hyperglycaemia detected for the first time at any point during pregnancy, with glucose values falling below those established by the WHO for diagnosing diabetes in adults [5]. Additionally, gestational diabetes is commonly identified during the second or third trimester of pregnancy.

Worldwide, there is a 16.7% incidence of hyperglycaemia in pregnancy, of which cases 80.3% are gestational diabetes [4]. In Spain, the Spanish Society of Gynaecology and Obstetrics offers similar data, with 87.5% of hyperglycaemia in pregnancy being caused by gestational diabetes. Furthermore, of all pregnant women, it is estimated that 12% or more, depending on the diagnostic strategy used, have gestational diabetes [6].

Gestational diabetes is caused by a deficit in insulin production in the pregnant woman, whose pancreas is unable to generate the insulin necessary to meet the insulin requirements of pregnancy. Normally, in the first trimester of pregnancy, there is a marked increase in insulin sensitivity, promoting glucose uptake by adipose tissue. However, as pregnancy progresses, placental hormones such as human chorionic gonadotropin (hCG), progesterone, oestrogen, and human placental lactogen (hPL) increase, many of which cause insulin resistance. In compensation, there is usually both increased pancreatic β-cell proliferation and reduced pancreatic β-cell apoptosis, so that the β-cell mass undergoes hypertrophy and hyperplasia, leading to increased insulin release, which maintains normal glucose levels. If β-cell dysfunction occurs, the compensatory effect is lost, resulting in gestational diabetes [7,8].

Understanding the pathophysiology of this disease, it is logical that screening for the disease is currently recommended between 24–28 weeks of gestation [9]. However, there is no international consensus on the advisability of earlier screening in early pregnancy, since authorities such as the American Diabetes Association, the National Institute for Health and Care Excellence, and the Spanish Society of Gynaecology and Obstetrics recommend earlier screening only for women with certain risk factors [6,9], and others, such as the International Federation of Gynecology and Obstetrics, recommend universal screening in early pregnancy, regardless of the presence or absence of risk factors [9].

There is also no consensus on the best screening and diagnostic strategy [10]. Traditionally, a two-step strategy has been used (50 g oral glucose overload, which, if greater than or equal to 140 mg/dL, is followed by a diagnostic test with a 100 g oral glucose overload). However, following the study “Hyperglycemia and Adverse Pregnancy Outcomes” [11], the International Association of Diabetes and Pregnancy Study Groups (IADPSG) and later the WHO recommended the one-step strategy [8,12], although the National Institute of Health does not support it [12], nor does the Spanish Society of Gynaecology and Obstetrics, which continues to recommend the two-step strategy currently used in Spain [6].

Having gestational diabetes poses risks for both maternal and foetal health, including an increased likelihood of macrosomia, birth injuries, respiratory problems, and neonatal hypoglycaemia, as well as a higher risk of preeclampsia. Women face an elevated risk of developing preeclampsia, as well as undergoing a caesarean delivery, which may contribute to heightened morbidity and, in severe cases, mortality. The extent of this elevated risk depends on factors such as the severity of preeclampsia and the specific circumstances surrounding the caesarean delivery. Much of these risks are related to the degree of glycaemic control during pregnancy, as the worse the control, the greater the risk of negative obstetric and neonatal outcomes, such as preterm birth, polyhydramnios, macrosomia, shoulder dystocia, increased admission to the neonatal intensive care unit, neonatal respiratory distress syndrome, foetal hypoglycaemia, and hyperbilirubinaemia. In addition, the risk of morbidity and mortality is also increased. In addition, women diagnosed with gestational diabetes have an increased risk of developing type II diabetes mellitus later in life [13].

Therefore, it is essential that good glycaemic control is achieved. The target glucose levels recommended by both the American Diabetes Association [14] and the Spanish Society of Gynaecology and Obstetrics [6] are fasting blood glucose < 95 mg/dL and one-hour postprandial blood glucose < 140 mg/dL or two-hour postprandial glucose < 120 mg/dL.

These target values are achieved primarily through dietary and lifestyle changes, which are the first line of treatment [6,8,9,12,13,14]. Between 70 and 85% of patients diagnosed with gestational diabetes can maintain normo-glycaemia with adequate physical activity and dietary and lifestyle modifications [14]. Thus, diet plays an essential role in the management of gestational diabetes. In the current scientific literature, a broad array of dietary approaches for managing gestational diabetes is documented. We formulated the following research question: What recent evidence exists regarding advancements in nutritional strategies for the management of gestational diabetes, and how effective are these strategies in enhancing maternal and foetal outcomes? Consequently, driven by this research question, our objective was to conduct a comprehensive review and synthesis of scientific evidence, aiming to update nutritional guidelines for the effective management of gestational diabetes.

## 2. Materials and Methods

### 2.1. Review Protocol

In order to achieve the proposed objectives, the methodology used was a systematic review of the scientific literature that has been published in the last five years on nutritional recommendations for the effective management of GDM. For this purpose, the Preferred Reporting Items for Systematic Reviews and Meta-Analyses (PRISMA) review guide of recommendations were followed, which is a list based on the verification of 27 items or points about the aspects of an original scientific work that are considered of greater relevance or representativeness. In addition, this guide also sets out the ideal preparation process that must be followed in order to produce a systematic review of both scientific and methodological quality.

This systematic review has been carried out following a protocol available on the website: http://www.crd.york.ac.uk/PROSPERO/ (accessed on 10 March 2023), with the registration number CRD42023423824.

### 2.2. Eligibility Criteria

Studies that met the following criteria were selected:

- Study design: only studies with a randomized clinical trial (RCT) methodology or design were eligible for selection.
- Year of publication: only studies published in the last five years were selected, i.e., with a publication date between 2019 and 2023; the establishment of this criterion allowed for an updated review of the topic addressed in the review.
- Study topic: only papers that could provide information about nutritional advice or recommendations to implement an adequate management of GDM were selected, excluding papers that dealt with the prevention of the disease, as well as those that did not deal with food as such (e.g., insulin treatments, metformin, etc.).
- Language of publication: no restriction was established, i.e., articles published in any language could be selected.
- Studies with a methodological quality on the PEDro scale [15] exceeding 5 points.

### 2.3. Sources of Information

The bibliographic search was carried out in electronic databases, specifically PubMed, the WOS Core Collection, SCOPUS, and CINAHL.

The structured language employed for conducting bibliographic searches in the selected electronic databases was obtained through the use of Medical Subject Headings (MeSH) and Health Sciences Descriptors (DeCS), both of which are presented below:

- MeSH: “Diabetes, Gestational”; “Nutrition Assessment (nutrition*)”; “Diet”; “Eating”; “Food”
- Natural Language: “Gestational Diabetes Mellitus”; “Diabetes Mellitus, Gestational”; “Diabetes, Pregnancy Induced”; “Gestational Diabetes”; “Diabetes, Pregnancy-Induced”
- DeCS: “Gestational Diabetes”; “Nutrition*”; “Diet”; “Food Intake”; “Food”

The Boolean operators used were “AND” and “OR”.

### 2.4. Search Strategy

The search strings or equations that have been designed are as follows:

▪ #1: (“gestational diabetes mellitus” OR “diabetes mellitus gestational” OR “diabetes, pregnancy induced” OR “gestational diabetes” OR “diabetes, pregnancy-induced” OR “diabetes, pregnancy induced”) AND (nutrition* OR diet OR eating OR food)
▪ #2: (“gestational diabetes mellitus” OR “diabetes mellitus gestational” OR “diabetes, pregnancy induced” OR “gestational diabetes” OR “diabetes, pregnancy-induced” OR “diabetes, pregnancy induced”) AND nutrition*
▪ #3: (“gestational diabetes mellitus” OR “diabetes mellitus gestational” OR “diabetes, pregnancy induced” OR “gestational diabetes” OR “diabetes, pregnancy-induced” OR “diabetes, pregnancy induced”) AND (nutrition* OR diet OR eating OR food) AND (RTC OR “randomized controlled trial”)
▪ #4: (“gestational diabetes mellitus” OR “diabetes mellitus gestational” OR “diabetes, pregnancy induced” OR “gestational diabetes” OR “diabetes, pregnancy-induced” OR “diabetes, pregnancy induced”) AND nutrition* AND (RTC OR “randomized controlled trial”)

Table 1 below shows the search strategy used to carry out the present review and the date on which the search process was carried out.

### 2.5. Data Extraction Process

Once the article search was conducted following the strategy described above, the selected articles were transferred to the Mendeley web application using the Mendeley Web Importer. Subsequently, the studies were organized into folders based on the electronic database from which they were collected, after which duplicate studies were removed.

For the compilation of this review, RCT studies were included that aimed to investigate how certain foods affect women with GDM for optimal disease management and were published between 2019 and 2023. The authors of this study examined the title, abstract, and keywords of each article collected during the search strategy, applying the study selection criteria in all cases. Additionally, in cases where articles were considered potentially eligible, the procedure was carried out in the same manner but, in this case, by analyzing the full text of the study.

Any possible doubts on the part of the study authors were resolved through discussion and final consensus with the study director. Likewise, data regarding the quality of the studies, as well as sample characteristics, intervention analysis, and even the most decisive results of the studies were obtained by the study authors.

### 2.6. Data Collection Process and Collected Data

A series of data was extracted from each study, deemed relevant, and exported to the results in Table 2. In this case, the extracted data from each study included: authors, year of publication, and country where the research was conducted; number and characteristics of participants (quantity, age, and diagnosis of the pathology, among others, depending on the eligibility criteria of the sample in each study); distribution of the sample into groups; characteristics of the intervention (gestational age at the start of the intervention, weight, BMI, objective of the intervention, assessment of food consumption, the intervention itself); results; and finally, the study’s conclusion.

### 2.7. Risk of Bias in Individual Studies

In order to methodologically assess the papers selected for this review, a design analysis was carried out. In this case, all were RCT-design studies, as established in the eligibility criteria during the reference search strategy. And for the assessment of the scientific quality of studies, it was considered relevant to use the PEDro scale (Table S1) as an evaluation instrument, which allows a score to be obtained based on a series of indicators (specifically 11), which are scored by adding 1 point (if they are present in the evaluated work) or 0 points (if they are not), such that total scores of 10 points can be obtained.

Thus, if the RCT achieves a total score of 9 or 10, it is considered of very good quality; if the score is between 6 and 8, the quality is good; scores between 4 and 5 indicate fair quality; and if the total score is below 4, the quality of the trial is considered inadequate. The results obtained from the scientific quality assessment of the 14 studies were selected for the development of this review (Table 2). In this case, as can be seen, the total scores of the studies range between 6 and 10, with a mean score of 9.6 ± 1.2, indicating that the mean scientific quality could be considered “good quality”.

## 3. Results

**Figure 1:**
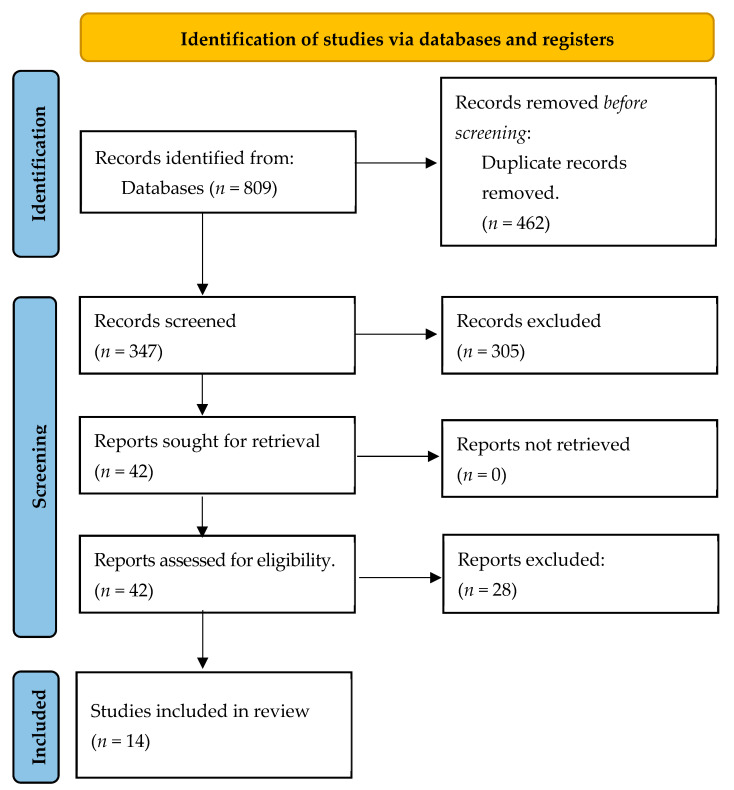
Flow diagram.

Figure 1 below shows the flow of selection of papers that took place until the studies that finally make up the review were obtained, following the proposal of the PRISMA Guide.

In this case, as shown in Figure 1, the total number of papers collected from the databases was 809, although 347 were left after eliminating duplicates. The title, abstract, and keywords of a total of 347 articles were evaluated, and 305 articles were excluded after this screening, leaving 42 papers for analysis of the full article. After this analysis, 28 were excluded for the reasons shown in Figure 1, and 14 papers were finally selected to form the present review.

As can be seen in Table 2, the aim of all the studies reviewed was to analyze how nutritional recommendations could be used to manage GDM effectively. And the results observed after the review indicate that, in general, personalized nutritional care for pregnant women with GDM as soon as possible after the diagnosis of the pathology has a positive influence on both maternal and neonatal outcomes, provided that they comply with the recommendations in an appropriate manner.

In summary, current evidence suggests that diet quality, personalized nutritional education, and dietary supplements such as probiotics, omega-3 fatty acids, antioxidants, and dietary fibre can have a positive impact on glycaemic control, weight gain, and other metabolic outcomes in pregnant women with GD. Supplementation should be individualized, taking into account each patient’s diet and adherence. Further high-quality randomized controlled trials are needed to formulate more specific and updated nutritional recommendations for the effective management of GD.

## 4. Discussion

It is worth starting by indicating that the main objective of the interventions reviewed is, in any case, to improve the quality of the diet consumed, and it has been observed that nutritional recommendations should be implemented in conjunction with the appropriate physical exercise in each case. Thus, even if the improvement in the quality of the diet is moderate or minimal, the efficacy in terms of improved maternal and neonatal outcomes is appreciable and positive in all cases [17,18,21,24,29].

More specifically, it has been determined that probiotic supplementation provides benefits to women with GDM [16,20], regardless of their intake protocol and composition, in a similar way to that proposed in previous studies, where improvement has been seen not only as a treatment for GDM, but also in the onset of the disease. In other words, probiotics, including well-known strains such as Lactobacillus acidophilus, Lactobacillus casei, Bifidobacterium bifidum, Lactobacillus fermentum, and the probiotic supplement Infloran, are proving their usefulness in recent years both therapeutically and preventively in the context of GDM in women [16,20,30,31,32].

Specifically, the present review found that a six-week supplementation in GDM patients with a probiotic capsule (LactoCare^®^, Zisttakhmir Company, Tehran, Iran) of Lactobacillus acidophilus, Lactobacillus casei, Bifidobacterium bifidum, and Lactobacillus fermentum, specifically with 2 ×10^9^ colony-forming units (CFU) of each bacterium per gram, has beneficial effects on the expression of genes related to insulin and inflammation, glycaemic control, some lipid profiles, inflammatory markers, and oxidative stress [16]. Specifically, a significant increase in gene expression of PPAR-γ, QUICKI, and HDL cholesterol levels and a significant reduction in FPG, insulin, HOMA-IR, triglycerides, VLDL cholesterol, and total cholesterol/HDL cholesterol were observed; however, probiotics did not affect the gene expression of LDLR and other lipid profiles [16].

Currently, few studies have investigated the effect of probiotics on insulin-related gene expression and lipid metabolism; however, there is some published information that may be related to this, such as the study by Chon et al. [33], where PPAR-γ polymorphisms were found to be highly correlated with the occurrence of GDM in pregnant women; therefore, probiotics, due to their beneficial actions on PPAR-γ, such as the probiotic capsule administered in the study by Babadi et al. [16], prove to be useful in controlling metabolic profiles in women with GDM.

On the other hand, although studies on the impact of probiotics on insulin-related gene expression and lipid metabolism are scarce, several studies have documented the beneficial effects of probiotics on glycaemic control and lipid profiles [20,30,31,32]. Similarly, a four-week intake of the probiotic supplement Infloran (SIT Pharmaceutical Laboratory, Mede, Italy, and imported by DKSH, Bangkok, Thailand), each capsule of which contained 1 billion CFU of Lactobacillus acidophilus and 1 billion CFU of Bifidobacterium bifidum, has also been shown to reduce fasting glucose in women with GDM and to increase insulin sensitivity [20].

Regarding the implementation of probiotics in the diet of women with GDM, it is also worth noting that in both studies reviewed where this has been investigated [16,20], Lactobacillus acidophilus and Bifidobacterium bifidum were present in the probiotic capsule, either exclusively [20] or together with other bacteria [16]. Today, it is still unclear which capsule composition is most effective, as well as the most recommended number of CFU. According to the data analyzed and reviewed, probiotics have a positive influence on glycaemic control and are a promising tool to reduce the frequency of GDM; however, there also seems to be consensus on the need for further studies to determine the optimal model of probiotic therapy (strain, dose, time of intervention, etc.) in pregnant women with GDM [30,31,32].

On the other hand, co-supplementation of vitamin D and probiotics has also been shown to be effective in terms of benefits for the metabolic status of these patients [19], in line with previous studies [34,35]. Specifically, in the study by Saha and Saha [35], joint supplementation with vitamin D and probiotics was found to decrease the risk of hyperbilirubinaemia in newborns (RR: 0.28; 95% CI: 0.09, 0.91), making vitamin D of great value beyond joint supplementation with probiotics in managing outcomes in GDM. Thus, in that study [35], it was concluded that vitamin D supplementation or co-supplementation in GDM patients showed a low burden of participant dropout and a low risk of caesarean section, newborn hyperbilirubinaemia, and newborn hospitalization.

On the other hand, the present review has also observed that a diet enriched with EVOO leads to a decrease in triglyceridaemia and weight gain, as well as having anti-inflammatory properties in the placenta and umbilical cord blood [22]. It should be noted that the study by Gómez Ribot et al. [22] has been proposed as the first to investigate the therapeutic effect of EVOO in women with GDM, so that the discussion of results can only be approached from a circumstantial angle rather than the comparison itself. It should be noted, first of all, that EVOO is the main vegetable oil that makes up the Mediterranean diet, which is increasingly considered a medical treatment [36,37].

It is also interesting to note that this Mediterranean diet has been found to be associated with a lower incidence of GDM [38], and it has also been observed that, in situations outside pregnancy, a diet enriched with EVOO has beneficial effects on metabolic and cardiovascular diseases [39]. Furthermore, the benefits of a diet enriched in EVOO and pistachios have also been demonstrated for the prevention of GDM in pregnant women [40]. Based on all these data, although there are no studies prior to the one reviewed [22] addressing the supposed beneficial effect of a diet enriched with EVOO as therapy in pregnant women with GDM, the results seem encouraging in this respect, but more research is needed to corroborate them and provide conclusive information.

On the other hand, the addition of oat bran also appears to be effective in terms of therapy in pregnant women with GDM according to one of the studies reviewed [25], with the beneficial impact concerning a decrease in mean fasting blood glucose and two-hour postprandial glucose observed at 2 and 4 weeks after the intervention [25]. However, these results cannot be compared and discussed with previous similar studies, as the work by Barati et al. [25] is the only study to date that has evaluated the effect of oat bran in cases of GDM.

However, the positive impact of oats on blood sugar in non-pregnant individuals and in patients diagnosed with type 2 DM has been analyzed and corroborated [41,42]. Therefore, based on the results of the study by Barati et al. [25] and the benefit demonstrated in other patient groups investigated [41,43], it is considered that implementing the addition of oat bran in the diet could be a possible effective recommendation in cases of women with GDM. However, further studies with larger sample sizes are recommended to test the efficacy of this valuable dietary supplement.

On the other hand, in the present review, it has been observed that the recommendations regarding carbohydrate intake are disparate, and not all interventions where diets have been designed to treat GDM during pregnancy with a specific focus on this type of biomolecules have been effective, despite the fact that carbohydrate intake is very relevant during pregnancy and GDM [43]. Such is the case of the proposal of Mijatovic et al. [23] in their work, where the aim was to reduce carbohydrate intake in women with GDM in order to understand its impact on blood ketone concentration, risk of ketonemia, and pregnancy outcomes in this group of women. In this study, the intervention to reduce carbohydrate intake in GDM did not raise ketones to clinical significance, with no differences in blood ketones as a function of higher or lower carbohydrate intake, although carbohydrate and total energy intake were significantly lower in the intervened women [23].

That is, despite lower energy intake, the work of Mijatovic et al. [23] could not detect any differences in pregnancy outcomes such as birth weight, gestational age at term rates, and % infant fat-free mass. Notably, these results were surprising, as some previous studies have reported that a higher glycaemic index diet and higher carbohydrate intake during the third trimester of pregnancy are recommendations associated with lower % fat-free mass and % fat mass, respectively [44].

Similar to the study by Mijatovic et al. [23] in terms of results that do not show a strong positive impact after a carbohydrate intervention in pregnant women with GDM is the work of Liu et al. [26]. In the latter, after ingestion of a low-concentration carbohydrate solution in the same group, in this case two hours prior to caesarean section, the results indicated that, although it is a safe intervention, the benefits were small for both mother and newborn [26]. However, although the benefits were small, it is noteworthy that it was observed that ingestion of the low-concentration carbohydrate solution two hours prior to surgery decreased the risk of hypoglycaemia, as the mean blood glucose level was slightly higher just prior to that induction of anaesthesia than that in women who did not undergo surgery. That is, the low-concentration carbohydrate solution had a positive effect on the prevention of hypoglycaemia before surgery [26], in agreement with previous studies [45,46].

On the other hand, also concerning carbohydrate intake in the setting of women with GDM, it is noteworthy that it has been observed that the intake of a high- or medium-carbohydrate snack before women go to bed appears to be associated with slightly higher fasting blood glucose levels in women with diet-controlled GDM than in women who do not take the above-mentioned snack [27]. In addition, glucose levels were found to be associated with an increased risk of adverse perinatal outcomes, as previously reported [11].

It is also interesting to note that data from the work of Henze et al. [27] did not support a significant reduction in fasting blood glucose levels in women when they snacked compared to women who did not snack, contrary to commonly given advice [11]. Increasing BMI was also found to increase fasting blood glucose levels, an effect that stabilized as BMI increased and has been previously observed [47]; however, associations with lower fasting blood glucose were also identified as gestation increased, which is surprising and for which there is a clear biological explanation, as insulin resistance has been found to increase significantly in the third trimester in women with type 1 diabetes and type 2 diabetes [48]. It is possible that patient-related bias or enhanced behaviours during the intervention could have had an effect on this outcome. The association between better sleep quality and lower fasting blood glucose in women with GDM [27] has been previously described [49].

Another diet that could be proposed as likely to have a beneficial impact on both mothers with GDM and their newborns is one that integrates nutraceutical supplements, specifically omega-3 fatty acids, anthocyanins, and alpha-cyclodextrins [29]. However, in the study by Soldavini et al. [29], no significant improvements were observed after the intervention in measurements of metabolic, inflammatory, or antioxidant parameters in blood and urine, which may be due, above all, to the differences being masked by the expected effect of the diet, highlighting its positive effects when multiple assessments of adherence and personalized advice to patients were carried out, as suggested in previous studies [50]. That is, adherence to the supplementation protocol was not adequate.

On the other hand, in addition to the supplementation reviewed in this research, it should be noted that some protocols or diets have also been identified that improve maternal outcomes in women with GDM and even in neonates in some cases. One of the interventions that seems to be effective is the one that recommends the intake of a diet with a higher percentage of complex carbohydrates and a higher percentage of fat (categorized as CHOICE diet) than the conventional one (60% and 15% versus 40% and 45%, respectively) has a beneficial effect on the maternal microbiome, as well as improving the diversity of the infant’s gut microbiome and reducing opportunistic pathogens that can play an important role in both obesity and immune system development [28].

Notably, this study by Sugino et al. [28] is the first study to compare maternal health and infant gut microbiome outcomes in a dietary intervention of two different dietary compositions (all meals provided) in women with GDM. Surprisingly, the Bifidobacteriaceae family of probiotics, specifically B, was found to be increased in the microbiota of women on the CHOICE diet. It should be noted that bifidobacteria are generally beneficial bacteria that attenuate intestinal inflammation and dysbiosis, inhibiting and reducing lipopolysaccharide-induced injury to the intestinal epithelium [51], as well as metabolizing resistant starches such as human milk oligosaccharides and other complex carbohydrates such as fructooligosaccharides and galactooligosaccharides [51]. Thus, the abundance of Bifidobacteriaceae B correlates with lower HbA1c and basal insulin requirements, suggesting an overall protective effect of the CHOICE diet on pancreatic B-cell function in women with GDM.

Among the limitations found in this review, it is generally observed that many intervened women do not comply with the recommendations of nutritional professionals in terms of recommended daily values of each element, and the intake of multivitamins is not recommended in many cases, which may condition the results of studies of nutritional interventions in women with GDM. In addition, small sample sizes and baseline research data such as BMI are also more likely to influence the results of interventions. Therefore, more studies with different food administration are needed to gain insight into the benefits and risks of low-carbohydrate diets, and personalized attention from nutrition professionals seems to be crucial to maximize the effectiveness of all proposed nutritional recommendations for women with GDM.

## 5. Conclusions

Among the different proposals being published in recent years to improve the outcomes of both the mother with GDM and her newborn, probiotic supplementation seems to be the most effective, both therapeutically and preventively. Specifically, supplementation with both the probiotic capsule LactoCare^®^ and Infloran is effective, as well as co-supplementation of probiotics with vitamin D; however, it is currently unknown which probiotic composition, quantity, and administration protocol might be most effective in women with GDM, and more research is needed. Other recommendations such as the implementation of a diet enriched with EVOO and the addition of oat bran to the diet appear to be other effective nutritional options as therapy in cases of GDM.

For their part, the results of carbohydrate intake interventions in women with GDM are controversial, partly because the objectives of the studies are diverse and therefore the results are not open to discussion and conclusions. Nevertheless, it appears that ingestion of a low-concentration oral carbohydrate solution two hours before caesarean section improves both maternal and neonatal outcomes. Similarly, the intake of a high- or medium-carbohydrate snack before bedtime by women with GDM also has a positive impact on fasting blood glucose levels.

And beyond the possible options for nutritional supplementation in women with GDM, diets designed specifically for this group of people are also being proposed, including the so-called CHOICE diet, which is categorized by a higher percentage of complex carbohydrates and lower fat content and has been found to have a beneficial effect on women. However, beyond the nature of the supplements and/or diets, one of the factors that seems to most influence their effectiveness is the adherence of women with GDM, and the need for specificity and individualized attention in each case seems crucial.

## Figures and Tables

**Table 1:** Search chain.

Source	Search Chain	Filters	Limits	Date	Outcomes
PUBMED	#1	RCT; Last 5 years		1 April 2023	177
	#2	RCT; Last 5 years		1 April 2023	95
WOS	#3	2019–2023	Abstract	15 April 2023	36
	#4	2019–2023	Abstract	15 April 2023	16
SCOPUS	#3	2019–2023	Article title, abstract, keywords	15 May 2023	324
	#4	2019–2023	Article title, abstract, keywords	15 May 2023	127
CINAHL	#3	2019–2023		4 May 2023	29
	#4	2019–2023		4 May 2023	5
TOTAL					809

**Table 2:** Table of results.

Author	Sample Characteristics (Inclusion Criteria)	Objective of the Intervention	Intervention	Conclusion
Babadi, Khorshidi, Aghdayood et al. [16], 2019. Iran.	*n* = 48 pregnant women. CG = 24; IG = 24. Age: 18–40 years. Exclusion criteria: participants with preeclampsia, eclampsia, thyroid disorders, smokers, with kidney or liver disease requiring initiation of insulin therapy during the intervention and taking probiotic products, including probiotic yogurt and kefir, during the intervention. Between 24 and 28 WG.	To assess the effects of probiotic supplementation on genetic and metabolic profiles in people with GDM not taking oral hypoglycaemic agents.	In the IG, patients received a probiotic capsule containing *Lactobacillus acidophilus*, *Lactobacillus casei*, *Bifidobacterium bifidum*, and *Lactobacillus fermentum* (2 × 10^9^ CFU/g each) for 6 weeks. Probiotic supplements and placebos (corn starch) were produced by LactoCare^®^, Zisttakhmir Company (Tehran, Iran), and were approved by the Food and Drug Administration.	Probiotic supplementation for 6 weeks in patients with GDM had beneficial effects on the expression of genes related to insulin and inflammation, glycaemic control, some lipid profiles, inflammatory markers, and oxidative stress.
da Silva et al. [17], 2019. Brazil.	*n* = 286 pregnant women. CG = 145; IG = 141. Age: 20 or more years at the time of conception. Diagnosed with GDM in the public maternity hospital in Rio de Janeiro between 2011 and 2014; pregnancy with a single foetus. Exclusion criteria: pregnant women with chronic diseases and with restrictive diets (vegetarian and others). Between 24 and 28 WG.	To compare the efficacy of nutritional counselling in GDM between the traditional method and the carbohydrate-counting method.	In a study on GD, participants received six individual appointments with a nutritionist during their pregnancy. They were provided with personalized guidance and a dietary plan based on their habits, socioeconomic status, and complications. Both groups (CG and IG) had similar diet plans, but the IG received carbohydrate-counting instructions with a list of foods grouped into 15 g carbohydrate servings. Follow-up appointments assessed compliance and adjusted recommendations as necessary.	Diet quality was associated with improved overall and postprandial glycaemic control in women with GDM. The results support the effectiveness of prenatal nutritional care for pregnant women with GDM, regardless of the method of dietary guidance applied. This suggests that nutritional care, including appointments with a nutritionist soon after GDM diagnosis, may have a positive impact on perinatal outcomes for these women.
Gadgil et al. [18], 2019. USA.	*n* = 1220 pregnant women. The sample was divided into four quartiles based on the HEI-2010 adherence scores. Age: 18 years or older (average 32 years). Diagnosed with GDM during a 12-month period between March 27, 2011, and March 30, 2012. Exclusion criteria: women who reported total energy intake, as <500 kcal/24 h (*n* = 14), or >3500 kcal/24 h (*n* = 51), 19 or had already given birth before the dietary evaluation (*n* = 94). Between 24 and 28 WG.	To investigate the possible association between diet quality and glycaemic control in women with GDM.	The intervention was carried out in two phases. The first was conducted by computer-assisted telephone interview, and the second was administered by mail and included detailed diet and physical activity questionnaires. Participants completed dietary measures and had at least one measure of fasting 1 h after breakfast and 1 h after lunch, and/or self-assessed capillary glucose 1 h after dinner during the 6 weeks after completion of the diet assessment.	The results indicated that even a small improvement in diet quality may be beneficial in achieving better glycaemic control in women with GDM, a fact of which clinicians should be aware.
Jamilian, Amirani, and Asemi [19], 2019. Iran.	*n* = 87 pregnant women. G1 (vitamin D + probiotics) = 30; G2 (probiotics) = 29; G3 (placebo) = 28. Age: 18–40 years; first pregnancy. Exclusion criteria: taking vitamin D supplements, probiotics, and/or synbiotics during the last 3 months prior to the intervention; insulin therapy during the intervention; preeclampsia; eclampsia; hypo- or hyperthyroidism, and smokers. Between 24 and 28 WG.	To assess the effects of co-supplementation of vitamin D and probiotics on metabolic profiles, biomarkers of inflammation and oxidative stress, and pregnancy outcomes in women with GDM.	Patients were randomly assigned to three groups to receive: G1 = vitamin D (50,000 IU/every 2 weeks) plus probiotic (8 × 10^9^ CFU/day) (n ¼ 30); G2 = probiotic (8 × 10^9^ CFU/day) (n ¼ 29); G3 = placebo, for 6 weeks.	Co-supplementation of vitamin D and probiotics had beneficial effects on metabolic status in women with GDM, as well as on some foetal parameters.
Kijmanawat et al. [20], 2019. USA.	*n* = 57 pregnant women. CG = 29; IG = 28. Inclusion criteria: singleton pregnancy, maternal age 18–45 years, normal foetal chromosomes or structures based on second trimester ultrasound and/or invasive prenatal diagnosis, and no history of chronic disease. Exclusion criteria: consuming probiotic food products (yogurt, fermented foods, and bean paste) within 2 weeks prior to research enrolment, as well as antibiotic exposure within 4 weeks prior to enrolment. Between 24 and 28 WG.	To assess the effect of probiotic supplements on insulin resistance in pregnant women with diet-controlled GDM.	Women received probiotic supplements containing Bifidobacterium and Lactobacillus (IG) or a placebo (CG), in a one-capsule format, daily after the morning meal for 4 consecutive weeks. Participants were advised to avoid probiotic-containing foods and supplements throughout the study period to minimize confounding from other probiotics. Participants were seen every 2 weeks in the antenatal clinic for standard antenatal treatment, follow-up of adherence to treatment guidelines, and follow-up of adverse effects of interventions. A three-day, 24 h dietary recall questionnaire was completed after 2 weeks of intervention and used as the participant’s representative diet during the study period.	After 4 weeks of probiotic supplementation in women with GDM controlled by diet at the end of the second and beginning of the third trimester of pregnancy, a reduction in fasting glucose and an increase in insulin sensitivity was achieved. Thus, probiotic supplements can be considered an adjunct treatment for glycaemic control in women with GDM.
Lv et al. [21], 2019. China.	*n* = 134 pregnant women. CG = 67; IG = 67. Inclusion criteria: singleton gestation, no metabolic disease and no liver or kidney dysfunction, no history of diabetes before pregnancy, no diabetes health education by nutrition professionals, and not given insulin.	To determine the effects of a nutritional nursing intervention based on glycaemic load (GL) for patients with GDM.	All pregnant women received personalized dietary counselling and a reasonable dietary plan; dietary management and exercise were designed for each case based on ideal weight, actual weight gain, and dietary habits. Patients were treated with insulin when blood glucose levels were not achieved. All pregnant women who were guided by the diet were advised to eat small meals 5–6 times a day, to avoid overeating, and to do an adequate amount of exercise. On the other hand, CG was assessed according to the traditional food exchange method and IG was assessed using the food exchange method based on the glycaemic concept. Fasting blood glucose and 2 h postprandial blood glucose were the parameters assessed in women after 2 weeks of intervention.	A GL-based nutrition nursing intervention was more effective than traditional nutrition nursing for GDM patients, and could effectively control blood glucose, reduce the incidence of pregnancy complications, and improve pregnancy outcome. Therefore, CG-based nutritional nursing intervention deserves to be popularized.
Gomez Ribot et al. [22], 2020. Argentina.	*n* = 45 pregnant women. G1 (control) = 15; G1 (GDM) = 15; G3 (GDM + EVOO) = 15. Exclusion criteria: BMI greater than 30 kg/m^2^ before pregnancy, multiple pregnancies, and concurrent pathologies, including thrombophilia, preeclampsia, pregestational diabetes, complications associated with chronic hypertension, anaemia with total haemoglobin below of 8 g/dL, and positive serology for HIV, VDRL, hepatitis B, or Chagas disease. Between 24 and 28 WG.	To know the effect of a diet enriched in extra-virgin olive oil (EVOO) on maternal metabolic parameters and placental proinflammatory markers in women with GDM.	In all three groups (G1: control; G2: GDM; G3: GDM-EVOO), women received dietary instructions to follow a nutritional plan with the following composition: 2100–2400 Kcal/day; carbohydrates 48–50%, proteins 18–20%, and lipids 30–32%. In G3 (EVOO intervention), EVOO was provided to improve adherence, and women were instructed to include three tablespoons of EVOO per day (36 g/day). The EVOO was to be consumed raw and at main meals. The group that did not receive the EVOO-enriched diet (G2) was instructed to include no more than one tablespoon of EVOO per day (0–12 g/day). Follow-up appointments with the obstetric and nutrition professionals were every 1–4 weeks, depending on the gestational age and needs of each woman. At subsequent visits, insulin was administered when blood glucose targets were not met.	A diet enriched in EVOO in patients with GDM reduced triglyceridaemia and weight gain; in addition, it had anti-inflammatory properties in the placenta and umbilical cord blood, possibly mediated by the regulation of PPAR pathways.
Mijatovic et al. [23], 2020. Australia.	*n* = 46 pregnant women. CG = 22; IG = 24. Age: 18–45 years (mean 33.3 years ± 0.6). Exclusion criteria: alcohol consumption; smokers; were on a gluten-free, vegetarian, or vegan diet; had had assisted reproduction; did not understand English; had major surgery in the previous 5 years; or other comorbidities in addition to obesity, hypertension, or dyslipidaemia. Between 24 and 32 WG.	To know the impact of a low-carbohydrate diet on the concentration of ketones in the blood, the risk of ketonemia, and pregnancy outcomes in women with GDM.	The low-carbohydrate diet (intervention; IG) targeted 135 g/d of absolute carbohydrate without energy restriction, based on the estimated average carbohydrate intake required during pregnancy. The CG diet targeted 180–200 g/d of absolute carbohydrate. The intake of less carbohydrate at baseline and more fibre-rich foods (especially among CG participants) was encouraged.	An intervention to reduce carbohydrate intake in GDM did not raise ketones to clinical significance, possibly because the target of 135 g/d was difficult to achieve during pregnancy. There was no difference in blood ketones between the groups with higher or lower carbohydrate intake, although carbohydrate and total energy intake was significantly lower in the intervened women than in those used as controls.
Yuan et al. [24], 2020. China.	*n* = 312 pregnant women. CG = 154; IG = 158. Inclusion criteria: women treated at the Fourth Hospital of Shijiazhuang between January 2014 and December 2016. Exclusion criteria: patients with acute complications due to pre-existing diabetes. Between 24 and 28 weeks of gestation.	To investigate the effect of 12 h comprehensive nutritional care on metabolism, blood glucose level, and neonatal birth weight.	The 12 h intervention carried out in the IG consisted of admitting the patients from 7:30 am to 7:30 pm accompanied by a nutritionist and a nursing professional. There, they provided meals for patients with gestational diabetes, guided exercise after the meals, and health education regarding nutrition, diabetes, and exercise. At the end of the 12 h, a WeChat group was created to maintain contact and resolve possible doubts from the patients.	Comprehensive 12 h nutritional care led to better glycaemic control and weight gain, improving both maternal and neonatal metabolic outcomes.
Barati et al. [25], 2021. Iran.	*n* = 112 pregnant women. CG = 56; IG = 56. Inclusion criteria: mobile, age 18–35, low blood sugar (fasting blood sugar equal to or greater than 92 mg/dL, hourly glucose change test equal to or greater than greater than 180 mg/dL, or blood sugar 2 h after consuming 75 g of glucose equal to or greater than 153 mg/dL), women at 24–28 weeks of gestation. Exclusion criteria: history of overt diabetes or a disease interfering with the research process (liver or kidney disease, mental illness, stroke, oat allergy, history of stillbirth, gestational diabetes, macrosome births, and family history of diabetes). Between 24 and 28 WG.	To assess the effect of oat bran consumption in women with GDM	Participants in both groups received a GDM diet. In addition to the diet, the IG received 600 g of oat bran (3 × 200 g packets, OAB™ from the Golden Light Cup Company). The IG women consumed 30 g (equivalent to 3 to 4 half tablespoons) of oat bran with lunch and dinner daily for four weeks. To follow up with the pregnant woman, a phone call was made every two nights as a consumption reminder, and phone calls were made to ask about any allergies to oats. Fasting glycaemia and 2 h post-fasting glycaemia were monitored at 2 and 4 weeks after the start of the intervention in both groups (3 cc of venous blood was taken after 8–12 h of fasting).	The addition of oat bran to the standard diet for pregnant women with GDM reduced fasting and 2 h postprandial blood glucose. However, further studies in this regard with larger sample sizes are recommended to test the efficacy of this valuable dietary supplement.
Liu et al. [26], 2021. China.	*n* = 85 pregnant women. CG = 42; IG = 34. Inclusion criteria: participants with scheduled caesarean section at a tertiary maternity hospital in Hangzhou, China, from January to December 2019. Age 18–45 years; patients with GDM, singleton pregnancy, estimated gestational age ≥ 37 weeks, activities of daily living with scores of 100 points, no hepatonephritic syndrome, no foetal abnormalities detected during antenatal check-up, no communication barriers, voluntary participation, and signing of written informed consent. Exclusion criteria: patient with a history of gastrointestinal disease or surgery, type 2 DM, severe surgical disease, poor blood sugar control during pregnancy, drug use, intrauterine foetal distress, newborn with congenital disease, unplanned preoperative intravenous energy supplementation, preoperative fasting time greater than 6 ± 0.5 h.	To investigate the safety and feasibility of taking a low-concentration carbohydrate solution 2 h before the induction of anaesthesia for patients with GDM.	Both groups received face-to-face preoperative education on details related to the surgery the day before surgery and had a solid diet 6 h before the operation. At 2 h before the induction of anaesthesia, the IG received a low-concentration carbohydrate solution (300 mL, 7.5% carbohydrate, 382.5 kJ total) that consisted of 22.5 g of carbohydrate. dissolved in 300 mL of water, while the CG received 300 mL of warm water. The participants had to finish the solution in 10 min. Lactated Ringer’s solution was administered intravenously at the time of surgery. All patients received 4 mg of ondansetron intravenously before anaesthesia and were fed a liquid diet 6 h postoperatively.	Ingestion of a low-concentration carbohydrate solution was safe and feasible for GDM patients undergoing caesarean section.
Henze et al. [27], 2022. Australia.	*n* = 68 pregnant women. G1 = 11; G2 = 10; G3 = 15; G4 = 12; G5 = 11; G6 = 9. Inclusion criteria: older than 18 years, between 24 and 34 WG, following consistent dietary patterns, and having had more than two fasting blood glucose measurements between 4.7 and 5.4 mmol/L in the week prior to recruitment. Exclusion criteria: need for insulin or other oral diabetes therapies as decided by the treating physician during the study, working shift work or irregular meal times, having had more than 4 days with fasting blood glucose levels ≥ 5.4 mmol/L in the week prior to recruitment, having a macrosomic foetus defined as abdominal circumference >90th percentile or polyhydramnios, requiring an interpreting service, having previously undergone bariatric surgery, having taken betamethasone, or requiring hospital admission during the study period.	To investigate the effect of different bedtime snacks (high carbohydrate) compared to the effect of lower-carbohydrate intake and compared to no snack at all on fasting blood glucose levels (in the morning) in women with diet-controlled GDM.	Participants completed three different phases in the intervention, each for 5 days. Women were asked to have a standardized upper-level snack at bedtime with carbohydrates for five days (phase 1), to have a standard low-carb snack at bedtime for five days (phase 2), and not have a bedtime snack for five days (phase 3). The highest-carbohydrate (GI1) snack consisted of a small jar of plain yogurt and a medium-small apple, providing 220 calories, 25 g carbohydrate, 10 g protein, 7.7 g fat, and 3.3 g fibre. The low-carbohydrate (GI2) snack consisted of 10 almonds and 20 g dark chocolate, providing 184 calories, 7.4 g carbohydrate, 4.2 g protein, 14.8 g fat, and 1.1 g fibre. Both snacks were low in glycaemic index.	Eating a snack (high or medium carbohydrate) before bedtime in women with GDM was associated with slightly higher fasting blood glucose levels in women with diet-controlled GDM than in women who did not eat the snack.
Sugino et al. [28], 2022. USA.	*n* = 34 pregnant women and 24 neonates. CG = 16; IG = 18. Regarding neonates: CG = 14 participants; IG = 10 participants. Age 20–36 years, BMI 26–39 kg/m^2^, singleton pregnancy, no significant comorbidities or obstetrics, no history of preterm delivery or preeclampsia, and GDM that could only be treated by diet. They also had to intend to breastfeed for at least the first 4 months. Exclusion criteria: meeting any criteria for overt diabetes, likely to fail the diet and requiring medical attention. Women taking beta-blockers, antihypertensives, or glucocorticoids, as well as smokers and non-English speakers, were excluded. In addition, maternal stool samples were excluded if they had taken antibiotics in the 4 weeks prior to sampling. Infant stool samples were excluded if their mother had received antibiotics at delivery, if they had taken antibiotics in the 4 weeks prior to stool sample collection, or if information on antibiotic consumption was missing at the time of sample collection. Between 24 and 28 WG.	To identify the pathological alterations in the intestinal microbiota of mothers with GD and their neonates.	The CG received a conventional diet (40% complex carbohydrates/45% fat/15% protein), and the IG received a CHOICE diet (60% complex carbohydrates/25% fat/15% protein). Pregnant women were provided with all meals, which were low in calories and similar in fibre content. At 30 and 37 weeks of gestation, an analysis was performed to observe the state of the intestinal microbiota. Neonates underwent analysis at 2 weeks, 2 months, and 4–5 months.	Overall, the study results suggested that an isocaloric GDM diet, containing more complex carbohydrates and less fat, has a markedly beneficial effect on the maternal microbiome, improves the diversity of the infant gut microbiome, and reduces opportunistic pathogens capable of playing a role in obesity and the development of the immune system.
Soldavini et al. [29], 2022. Italy.	*n* = 40 pregnant women. CG = 23; IG = 17. Age: 18 years or older. Exclusion criteria: multiple pregnancies, foetal malformations, maternal diseases (type 1 or 2 diabetes, hypothyroidism or hyperthyroidism, immunological disorders), and abnormal blood glucose values before 24 weeks of gestation. Between 24 and 28 weeks of gestation.	To evaluate the effect of nutraceutical supplements (omega-3 fatty acids, anthocyanins, and alpha-cyclodextrins) in patients with GDM and to evaluate the role of anthropometric, metabolic, and inflammatory parameters as biomarkers to identify subjects who require hypoglycaemic drug treatment during pregnancy.	The women received nutrition education and a personalized diet by an expert educator based on references to the standard Mediterranean diet, the healthy eating recommendations of the Harvard University School of Public Health, and reference levels of dietary intake of nutrients and energy for pregnant women of the Italian population. The total daily energy intake was distributed over three main meals (breakfast, lunch, and dinner) and two snacks. Caloric intake was calculated according to the mother’s BMI and pre-pregnancy weight gain. Macronutrient composition was balanced as follows: 45% of total energy from carbohydrates, with simple sugar less than 12%; 25–35% total energy from fat (less than 7% saturated fat and 10% PUFA). Protein intake was that required in pregnancy according to the recommendations for the Italian population, with 50% protein of vegetable origin and 50% of animal origin. The quality of protein intake was regulated by the following consumption frequencies: meat, preferably white, 2 times/week; fish, 2–3 times/week, with a preference for oily fish for optimal intake of omega-3 fatty acids; legumes, 3–4 times/week; eggs, 2 times/week; cheese, 1 or 2 times a week; ham, once a week; nuts, 20–30 gr every day. High-glycaemic-index foods were not allowed. Two fruits and three servings of vegetables per day were recommended. Olive oil was indicated as the main culinary lipid. Dietary cholesterol was less than 200 mg/day and fibre intake was about 30 g/day. Anti-inflammatory nutraceutical supplements included the following: (1) omega-3 fatty acids (tablets, EnerZona Omega3Rx^®^, Enervit, Milano, Italy) at a daily dose of 2.4 g at breakfast; (2) anthocyanins (pills, EnerZona Maqui Re-sponse Capsule^®^, Enervit, Italy) at a total daily dose of 108 mg divided into three equal doses at breakfast, lunch, and dinner; (3) alpha-cyclodextrins (sachets, EnerZona Maqui Response Buste^®^, Enervit, Italy) at a total daily dose of 15 g divided into three equal doses at breakfast, lunch, and dinner.	Woman-to-woman nutritional coaching and low compliance with nutraceutical supplementation could have outweighed the impact of this intervention. Despite this, the concentration of triglycerides and the AA/EPA ratio seemed to be a biomarker of increased inflammation and GD in candidates for drug treatment. In addition, adequate administration of omega-3 in women with GDM, either by controlled diet or nutraceutical supplementation, reduced the need for drug therapy.

## Supplementary Materials

The following supporting information can be downloaded at: https://www.mdpi.com/article/10.3390/jcm13010037/s1, Table S1: Methodological assessment according to the PEDro scale.
