# Supplementary material for: Advancements in Nutritional Strategies for Gestational Diabetes Management: A Systematic Review of Recent Evidence
Source: J Clin Med. 2023 Dec 20;13(1):37. doi: 10.3390/jcm13010037 (PMC10779518; doi:10.3390/jcm13010037)
Supplement: Supplementary file 1 [file jcm-13-00037-s001.zip › jcm-2738519-supplementary.pdf]

Table S1. Methodological assessment according to the PEDro scale.

AUTHOR (YEAR)	TITLE	SCORE
Babadi et al. (2019) [16]	The Effects of Probiotic Supplementation on Genetic and Metabolic Profiles in Patients with Gestational Diabetes Mellitus: a Randomized, Double-Blind, Placebo-Controlled Trial.	10
Gabriel da Silva et al. (2019) [17]	Food intake of women with gestational diabetes mellitus, in accordance with two methods of dietary guidance: a randomised controlled clinical trial.	10
Gagdil et al. (2019) [18]	Dietary Quality and Glycemic Control Among Women with Gestational Diabetes Mellitus.	6
Jamilian, Amirani and Asemi (2019) [19]	The effects of vitamin D and probiotic co-supplementation on glucose homeostasis, inflammation, oxidative stress and pregnancy outcomes in gestational diabetes: A randomized, double-blind, placebo-controlled trial.	10
Kijmanawat et al. (2019) [20]	Effects of probiotic supplements on insulin resistance in gestational diabetes mellitus: A double-blind randomized controlled trial.	10
Lv et al. (2019) [21]	Effects of nutritional nursing intervention based on glycemic load for patient with gestational diabetes mellitus.	10
Gómez Ribó et al. (2020) [22]	An extra virgin olive oil-enriched diet improves maternal, placental, and cord blood parameters in GDM pregnancies.	8
Mijatovic et al. (2020) [23]	Effects of a modestly lower carbohydrate diet in gestational diabetes: a randomized controlled trial.	10
Yuan et al. (2020) [24]	A 12-hour comprehensive nutrition care benefits blood glucose level and weight gain and improves outcomes in pregnant women with gestational diabetes mellitus.	10
Barati et al. (2021) [25]	The effect of oat bran consumption on gestational diabetes: a randomized controlled clinical trial.	10
Liu et al. (2022) [26]	Safety and feasibility of oral carbohydrate consumption before cesarean delivery on patients with gestational diabetes mellitus: A parallel, randomized controlled trial.	10
Henze et al. (2022) [27]	The effect of bedtime snacks on fasting blood glucose levels in gestational diabetes mellitus.	10
Sugino et al. (2022) [28]	A maternal higher-complex carbohydrate diet increases bifidobacteria and alters early life acquisition of the infant microbiome in women with gestational diabetes mellitus.	10
Soldavini et al. (2022) [29]	Maternal AA/EPA Ratio and Triglycerides as Potential Biomarkers of Patients at Major Risk for Pharmacological Therapy in Gestational Diabetes.	10
